# CAR links hypoxia signaling to improved survival after myocardial infarction

**DOI:** 10.1038/s12276-023-00963-9

**Published:** 2023-03-20

**Authors:** Fabian Freiberg, Meghna Thakkar, Wiebke Hamann, Jacobo Lopez Carballo, Rene Jüttner, Felizia K. Voss, Peter M. Becher, Dirk Westermann, Carsten Tschöpe, Arnd Heuser, Oliver Rocks, Robert Fischer, Michael Gotthardt

**Affiliations:** 1 Translational Cardiology and Functional Genomics, Max Delbrück Center for Molecular Medicine in the Helmholtz Association, Berlin, Germany; 2 DZHK (German Centre for Cardiovascular Research), partner site Berlin, Berlin, Germany; 3 Department of Cardiology, Charité-Universitätsmedizin Berlin, Berlin, Germany; 4 Department of General and Interventional Cardiology, University Heart Center Hamburg Eppendorf, Hamburg, Germany; 5 DZHK Partner Site Hamburg/Kiel/Lübeck, Hamburg, Germany; 6 BCRT (Berlin-Brandenburg Center for Regenerative Therapies), Berlin, Germany; 7 Animal Phenotyping, Max Delbrück Center for Molecular Medicine in the Helmholtz Association, Berlin, Germany; 8 Spatiotemporal Control of Rho GTPase Signaling, Max Delbrück Center for Molecular Medicine in the Helmholtz Association, Berlin, Germany

**Keywords:** Translational research, Experimental models of disease, Heart failure, Mechanisms of disease

## Abstract

The coxsackievirus and adenovirus receptor (CAR) mediates homo- and heterotopic interactions between neighboring cardiomyocytes at the intercalated disc. CAR is upregulated in the hypoxic areas surrounding myocardial infarction (MI). To elucidate whether CAR contributes to hypoxia signaling and MI pathology, we used a gain- and loss-of-function approach in transfected HEK293 cells, H9c2 cardiomyocytes and CAR knockout mice. CAR overexpression increased RhoA activity, HIF-1α expression and cell death in response to chemical and physical hypoxia. In vivo, we subjected cardiomyocyte-specific CAR knockout (KO) and wild-type mice (WT) to coronary artery ligation. Survival was drastically improved in KO mice with largely preserved cardiac function as determined by echocardiography. Histological analysis revealed a less fibrotic, more compact lesion. Thirty days after MI, there was no compensatory hypertrophy or reduced cardiac output in hearts from CAR KO mice, in contrast to control mice with increased heart weight and reduced ejection fraction as signs of the underlying pathology. Based on these findings, we suggest CAR as a therapeutic target for the improved future treatment or prevention of myocardial infarction.

## Heart attack: Inhibiting cell contact protein may limit damage

During a heart attack the death of heart muscle cells causes lesions that transform into fibrotic tissue. Restricting the damage is critical, as mamalian heart muslce does not regenerate. The cell contact protein CAR, is upregulated in tissue surrounding lesions, but it has remained unclear if this part of the pathology or an attempt to contain the lesion. Michael Gotthardt at the Max-Delbrück-Center for Molecular Medicine in Berlin, Germany, and co-workers, examined CAR’s role following heart attack in CAR-inactivated and control mice. CAR exacerbates responses to low oxygen conditions and boosts a protein involved in cell death. The hearts of CAR-inactivated mice were not as enlarged as those of controls, and lesions were smaller and less fibrotic. Importantly, CAR deficiency improved survival, suggesting CAR inhibition as a suitable therapeutic strategy for patients with myocardial infarction.

## Introduction

Cardiovascular disease is among the most common causes of death and ischemic heart disease is the leading contributor to the disease burden^1^. The capacity for regeneration of cardiomyocytes in the mammalian heart is modest, even after overexpression of cell cycle regulators, limiting recovery after myocardial infarction (MI)^2^. Thus, the mammalian heart has to partially compensate for the loss of cardiomyocytes with non-muscle cells and proteins of the extracellular matrix (ECM)^3^. This process is tightly regulated and depends on the balance of diverse cardiac signaling pathways^4,5^. Although the pathways and cells involved in scar formation have been studied in detail, it is still unclear how lesion size, protein composition and connection to the healthy myocardium by cell contact proteins are shaped to balance stability versus dynamic properties and improve cardiac function post-MI.

A prominent cell contact protein that interacts with multiple proteins of the cardiac extracellular matrix is the coxsackie and adenovirus receptor (CAR)^6^. In the heart, this receptor not only mediates virus uptake^7,8^ but is also important in early cardiac development^9,10^ and electrical coupling in the adult heart^11,12^. CAR is a type 1 transmembrane protein with two extracellular IG domains that interact with virus, matrix, and other cell contact proteins (including CAR itself). Its cytoplasmic tail is linked to endocytosis and signal transduction pathways^13^. CAR expression is tightly regulated, with high expression levels in the developing heart and brain and decreased expression after birth^14–16^. It’s expression is reinduced upon cardiac remodeling, as observed in human dilated cardiomyopathy^17^, after induction of autoimmune myocarditis^18^ or MI in the rat^15^. In the latter model, CAR is specifically upregulated in areas surrounding the lesion, suggesting a role in local remodeling. Nevertheless, it is unclear whether the upregulation of CAR is beneficial, e.g., by containing the lesioned area or improving remodeling versus a contribution of CAR to the underlying pathology. This distinction has important implications, as future CAR-directed therapies would require diametrically opposed strategies depending on CAR’s role in MI.

Since HIF-1α signaling plays a major role in the response to hypoxia and as GTPase signaling pathways with RhoA and ROCK kinase were both linked to apoptosis and ischemic damage before^19^, we hypothesized that these pathways might be involved in the signaling processes connecting CAR expression and hypoxia-induced cell damage upon ischemia.

We addressed the role of CAR in hypoxia signaling with gain-of-function experiments in cell culture combined with a loss-of-function approach in our inducible heart-specific CAR-knockout mouse to evaluate its role in MI^12^. We found that depleting CAR from cardiomyocytes improved survival, cardiac remodeling, and contractile function and that the underlying mechanism relates to a role of CAR in regulating cellular survival via RhoA, p38 and HIF-1α. Together, these findings identify a role of CAR in the pathogenesis of ischemic heart disease and suggest CAR inhibition as a therapeutic approach for the future treatment of MI.

## Materials and methods

### Experimental design

The objective of this study was to test the hypothesis that CAR can improve cardiac function after MI by containing the lesion and improving remodeling. We used an in vivo loss-of-function approach with coronary artery ligation in inducible heart-specific CAR knockout mice. In a complementary gain-of-function approach, we overexpressed CAR-Cherry vs. Cherry only in HEK293 cells and mCAR in rat H9c2 (2-1) myoblast cells treated with oxygen deprivation or chemical hypoxia.

### Animal experiments

All experiments involving animals were carried out following the Guide for the Care and Use of Laboratory Animals of the German animal welfare act, and protocols were approved by the Committee on the Ethics of Animal Experiments of Berlin State authorities (LaGeSo). The generation of heart-specific inducible CAR knockout animals (strain name: Tg(MerCreMer) CXADR^tm1Mgot^) and induction with tamoxifen hav e been described previously^12^. In this strain, CAR protein levels are reduced to <20% after one week of tamoxifen injections, consistent with a protein half-life of <3 days. We only used male animals with tamoxifen injections starting at 7 weeks of age. The investigator was not blinded.

### Myocardial infarction

For the occlusion surgery, a published procedure was followed^20^. In brief, animals were anesthetized with xylazine/ketamine (5 [mg/kg BW]/100 [mg/kg BW], i.m.), intubated and respirated mechanically. After left thoracotomy, the pericardium was carefully removed. The left coronary artery was ligated 1 to 2 mm below its origin. Successful ligation was monitored by evaluating the macroscopic changes and recording ECGs. For the control treatment, the suture was not closed (sham surgery). For the wild-type control, we used Cre-negative animals treated with tamoxifen. Animals that died during the procedure were not included in the analysis.

### Echocardiography (ECG)

For echocardiography, we used the Vevo 770 system (Visual Sonics, Inc.) with a 45 MHz transducer mounted on an integrated rail system. Standard imaging planes and functional calculations were obtained according to the American Society of Echocardiography guidelines. The LV parasternal long axis 4-chamber view was used to derive fractional shortening (%FS), ejection fraction (%EF), and ventricular dimensions and volumes. Cardiac output (CO) was calculated using the formula heart rate = (stroke volume * heart rate)/1000.

### Histology

Aseptically removed tissues were fixed for 12 h in phosphate-buffered saline (pH 7.2) with 4% paraformaldehyde and embedded in paraffin. Histological analysis was performed on deparaffinized 5 µm sections stained with H&E or Masson’s trichrome to visualize the degree of fibrosis. For quantification, we used ImageJ.

### H9c2 cell culture and cardiac differentiation

H9c2 myoblast cells were maintained in DMEM with 10% FBS (fetal bovine serum) and 1% penicillin/streptomycin at a confluence lower than 70–80%. These cells were transfected with pCMV mouse CAR plasmid (2000 µg, mCAR) and Pei 160 reagent or untransfected as a control. Then, H9c2 mCAR-transfected cells were selected under 400 µg/ml Zeocin for 3 weeks. Cardiac differentiation was carried out in cells with a confluence higher than 80% with 1% FBS and 1 µM RA (retinoic acid, Focus Biomolecules) for 9 to 10 days before chemical hypoxia.

### Induction of hypoxia in cultured cells

For physical hypoxia, cell culture dishes with HEK 293 cells were placed inside a modular incubator chamber (Billups Rothenberg) that was prewarmed and humidified using a petri dish with sterile water (>30 min at 37 °C). For removal of O_2_, the chamber was flushed with 95% N_2_ and 5% CO_2_, disconnected and returned to the incubator at 37 °C for 24 h. For chemical hypoxia, 50 µM, 200 µM or 500 µM CoCl_2_ (Sigma-Aldrich) was added to the medium. H9c2 cells were treated with 500 µM CoCl_2_ for 24 h.

### Transcription factor activity assays

HEK 293 cells were seeded onto 24-well plates and transfected for 48 h using Pei 40 reagent and pCMV mCherry C1 (800 ng) and TA Luc (100 ng) or TF Luc (100 ng) for controls vs. CAR Cherry (800 ng) and TA Luc (100 ng) or TF Luc (100 ng), each plus 100 ng of internal control (RL-TK, Promega). After 24 h of hypoxia treatment with CoCl_2_ or 95% N_2_ + 5% CO_2_, we measured reporter activity using the Dual Luciferase Reporter Assay System (Promega) according to the manufacturer’s instructions. Light units of firefly luciferase were normalized to Renilla luciferase activity to determine relative luciferase units (RLU). Relative fold change (FC) was calculated using untreated controls with Cherry expression plasmid as a reference.

These experiments were performed in biological quadruplets with at least one independent replicate. The graphs represent one experiment. The values of the individual experiments were not pooled due to the differences in the internal control (Renilla) signal of each run that depends on factors such as temperature and batch of the substrate.

### Detection of ROS

Intracellular ROS were detected using Total Ros/Superoxide Detection kit (Enzo Life Sciences-Catalog # 51010). 48 h post transfection (24 h after CoCl_2_ treatment), HEK 293 cells were trypsinized, washed with 1X wash buffer and re-suspended in 1X ROS wash buffer with Oxidative stress dye at concentration of 1:1250 (300–500 µl) along with the inducer (positive control) or vehicle (test samples) for 30 min at 37 °C in the dark. Negative controls were treated with the inhibitor 30 min prior to inducer treatment in 1X wash buffer. Fluorochrome-loaded cells were excited using a 488 nm argon-ion laser in a flow cytometer (FACSort, BD). The emission of oxidative stress dye (total ROS) was measured at 520 nm. Data from at least 10,000 cells were collected for analysis. The mean fluorescence intensity (MFI) was calculated from cells that expressed Cherry or CAR fused to Cherry after gating for Cherry-positive cells. These experiments were performed in biological quadruplicates and repeated at least twice.

### Cell viability assay

The CellTiter-Glo reagent from Promega was used to perform a cell viability assay in HEK 293 cells 48 h after transfection (24 h after CoCl_2_ treatment). Twenty microliters of RT-equilibrated Cell Titer Glo reagent mix was used per 100 µl of media per 96-well. This 96-well plate was kept on a shaker for 5 min followed by 30 min of incubation in the dark at RT. Luminescence was recorded using a Tecan Infinite M200 plate reader with an integration time of 0.5 s per well.

### Protein extraction

Left ventricles with the septum from CAR wild-type and knockout mice were harvested and snap frozen. With pestle and mortar, the tissue was ground into coarse granules on dry ice and divided into three similar fractions. The ground tissue was further homogenized in 150-200 μl of 1x RIPA buffer (50 mM Tris pH 8; 150 mM NaCl; 1% NP-40; 0.1% Na-DOC; 5 mM EDTA; 0.1% SDS including protease and phosphatase inhibitors and 20% glycerol) using Ultra-Turax (IKA). For cultured cells, we scraped cells in 1x RIPA buffer and incubated the samples for an additional 30 min at 4 °C, followed by sonication and centrifugation to remove debris. We determined the protein concentration using the micro BSA kit protocol from the Pierce™ BCA Protein Assay Kit.

### WES

For quantitation of proteins by capillary electrophoresis, we used the Simple Western System (WES, Simon™, ProteinSimple, San Jose, CA). Wes™ reagents were obtained from the manufacturer and used according to the manufacturer’s recommendations with minor modifications. Protein lysates were adjusted to working stocks of 2 mg/ml and diluted using 0.1X sample buffer and 5X master mix (200 mM DTT, 5× sample buffer, 5× fluorescent standards), denatured at 95 °C for 5 min and run together with fluorescent standards. Proteins of interest were identified using specific primary antibodies diluted with Protein Simple antibody diluent. Samples were loaded onto the designated wells in a microplate prefilled with separation gel and subjected to electrophoresis (375 V, 31 min, 25 °C) and immunodetection following the manufacturer’s instructions. Data analyses was performed using the Compass Software (ProteinSimple). The area under the curve of the protein of interest was divided by the area under the curve of the loading control protein peak (Vinculin or GAPDH). Electrophoretic images were automatically generated by the Compass Software.

### Expression analysis

RNA from the indicated tissues was amplified with specific TaqMan probes. The probe sets used were as follows: CAR: AGCTGCACGGTTCAAAACAGA (forward), TTCCGGCTCGGTTGGA (reverse), 6-FAM-CTCTGACCAGTGTATGCTGCGACTAGACGT-TAMRA (probe). Probes for Connexin 43, Connexin 45, HIF-1α, and the VIC/TAMRA assay for 18S RNA were purchased from Applied Biosystems (Foster City, California).

### Rho-GLISA assay

We used the RhoA activation assay kit (Cytoskeleton, Inc.) as suggested by the manufacturer. Transfected 293 cells were washed with cold PBS, harvested in lysis buffer (with protease inhibitor cocktail) provided by the company and adjusted to 0.5 mg/ml protein. Appropriate positive and negative controls were used as indicated in the protocol, and samples were run in duplicate to detect Rho-GTP (active).

### Rhotekin pulldown assay

The assay was adapted from earlier work with minor changes^21^. Transfected HEK 293 cells were harvested in M2 lysis buffer (50 mM Tris HCl, pH 7.4, 150 mM NaCl, 10% glycerol, 1% Triton X, 0.5 mM EDTA, 0.5 mM EGTA and protease inhibitor (Roche Applied Science)) and sonicated. After removal of debris by centrifugation, the supernatant was divided into equal fractions for testing, positive control (addition of GTPɣs, 0.2 mM final) and negative control (addition of GDP 1 mM final) and incubated for 15 min at RT. GTP loading or unloading was stopped using 6 mM MgCl_2_ followed by incubation with 30 μg of RDB beads for 1 h at 4 °C (kind gift from Enno Klussmann). Beads were washed two times with cold M2 lysis buffer, resuspended in 4x SDS‒PAGE sample buffer (30% glycerine; 3% SDS; 200 mM Tris-HCl; 30 mM DTT; pH 6.8), and analyzed by SDS‒PAGE.

### Illumina gene expression profiling

RNA from left ventricles was isolated and purified using RNeasy columns (Qiagen) and stored at –80 °C. To reduce ribosomal RNA (rRNA), we used the RiboMinus Kit from Invitrogen (Carlsbad, CA). Biotinylated cRNA was prepared using the Illumina® TotalPrep™-96 RNA Amplification Kit (Ambion, Inc., Austin, TX) according to the manufacturer’s instructions with 100 ng total RNA. Expression profiling was performed using Illumina MouseRef-8 v2.0 Expression BeadChip, quantile normalized without background subtraction. Genes with an expression value of less than 0.05 in all samples were retained for subsequent analysis. Gene Ontology (GO) clustering was performed using the Cytoscape 3.2.1 plugin ClueGO v2.1.7^22,23^ on microarray data filtered for expression values below 0.05. We converted the gene names to UniProt ID annotations using the web-based application DAVID^24^. We used the following ontologies: biological processes, molecular function and wiki pathways. The enrichment/depletion statistics were performed using Bonferroni’s posttest *p* value correction for multiple testing. The kappa score was set to 0.4.

### Cardiomyocyte culture and hypoxia experiments

Adult ventricular cardiomyocytes were prepared from wild-type and heterozygous CAR KO mice^12^ at the age of 10–12 weeks by enzymatic dissociation as described previously^25^. Cardiomyocytes were seeded at a density of 7000 cells/ml in 24-well plates in murine myocyte culture medium (minimum essential medium containing bovine serum albumin, 0.1%, 2,3-butanedione monoxime, 10 mM, insulin-transferrin selenium supplement, 1x, penicillin/streptomycin, 1% and L-Glutamax, 1:100). After 1 h of settling, the medium was changed, and with this method, we routinely obtained a high percentage (>80%) of rod-shaped, viable cardiomyocytes. Before application of CoCl_2_ and after 24 h, 10 pictures were taken from each well to count the rod-shaped and dead cardiomyocytes to calculate the cell viability (cell viability (%) = (viable cells * 100)/total number of cells).

### Statistics

For statistical analysis, GraphPad Prism software 7.0 (San Diego, California) was used. The results are expressed as the mean ± SEM. For hemodynamic data, statistical significance between groups was determined using the Mann‒Whitney U test. For expression analysis, we used an unpaired two-tailed *t*-test. Asterisks indicate statistically significant differences (*P < 0.05; **P < 0.01; ***P < 0.001; ****P < 0.0001).

## Results

**Fig. 1:**
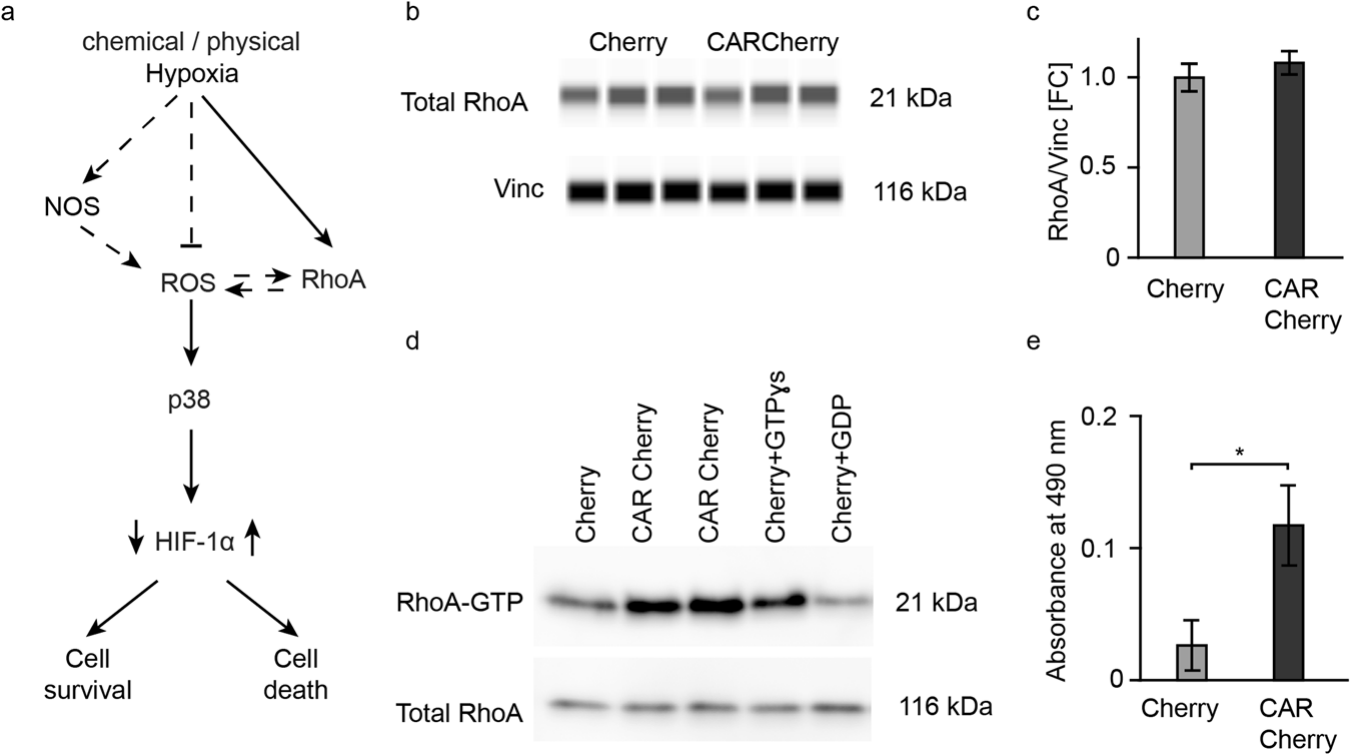
Increased RhoA activation in cells overexpressing CAR. **a** Schematic of the hypoxia-related signaling cascade influencing cell survival upon an ischemic event. **b** WES blot and **c** quantification of total RhoA in cells overexpressing Cherry vs. CAR Cherry. **d** Rhotekin pulldown assay: Representative western blot of the eluent from Cherry (Lane 1), CAR Cherry (Lanes 2-3), positive control (Lane 4), and negative control (Lane 5). **e** Independent quantification of active RhoA (G-LISA RhoA activation assay kit). Unpaired Student’s *t* test (*n* = 3) *p < 0.05.

To investigate a possible connection between CAR expression and the RhoA pathway and hypoxia signaling via HIF-1α and its downstream targets (Fig. 1a), we used HEK 293 cells and H9c2 cardiomyocytes. After transfection with CAR-Cherry vs. the Cherry control or CAR vs. the untransfected control, we treated these cultures with chemical hypoxia (24 h application of 50 µM or 500 µM CoCl_2_ as a hypoxia-mimetic agent) or physical hypoxia (95% N_2_ + 5% CO_2_ as a hypoxic stimulus).

### CAR acts upstream of the RhoA/ROS/HIF-1α signaling pathway to regulate apoptosis

We found that RhoA protein levels were not altered in the cells overexpressing CAR (Fig. 1b, c). Therefore, we evaluated RhoA activity using the Rhotekin pulldown assay^21^, where Sepharose beads are immobilized with the Rho effector Rhotekin binding domain (RBD), which specifically binds only to GTP-bound RhoA (active RhoA)^26^. RhoA activity was increased in the cells overexpressing CAR compared to the Cherry-overexpressing cells (Fig. 1d (Lanes 1–3)). The specificity of the beads to detect active RhoA was validated by adding GTPγs to load RhoA with a high amount of GTP as a positive control (Fig. 1d, Lane 4). As a negative control, we used lysates from the Cherry-transfected samples with GDP to favor the hydrolysis of GTP-bound RhoA (Fig. 1d, Lane 5). To validate these findings with an independent and quantitative approach, we used the Rho-ELISA kit. Equal amounts of protein lysates were incubated on plates coated with GTP-bound RhoA-specific substrate, and the captured RhoA-GTP was detected by ELISAs and quantified using spectrophotometry at 490 nm, documenting the increased RhoA activity in cells overexpressing CAR (Fig. 1e).

**Fig. 2:**
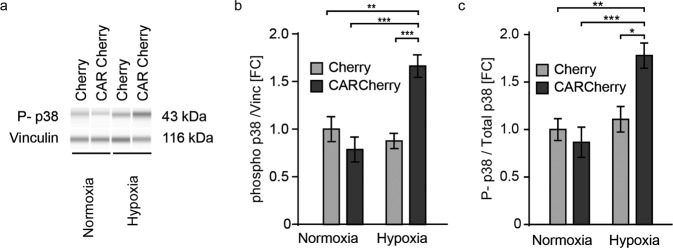
Activation of p38 during hypoxia is CAR dependent. Cells overexpressing Cherry or CAR Cherry were cultured under normoxia or hypoxia for 24 h. **a** Representative blot of capillary gel electrophoresis. **b** Quantification of phospho-p38 normalized to vinculin levels. **c** Quantification of phospho-p38 normalized to total p38. Two-way ANOVA with Bonferroni’s post-test. ***p < 0.001, **p < 0.01 and *p < 0.05 (*n* = 6).

As Rho levels and the generation of reactive oxygen species (ROS) affect each other and the regulation of ROS serves as a feedback loop to maintain O_2_ homeostasis within cells, we also investigated whether CAR affects total ROS levels in cells (Supplementary Fig. 1). Using a flow cytometry-based method (FACS) with an oxidative stress detection reagent (Enzo Life Sciences), we exposed CAR Cherry- and Cherry-transfected cells to hypoxia and analyzed them as described below. In the CAR-overexpressing cells, mean fluorescence intensities (MFIs) were increased compared to those of the control cells, indicating higher ROS levels in the presence of CAR. Both treatment with 50 μM CoCl2 and overexpression of CAR resulted in elevated ROS levels. The effect was additive with a stronger contribution of CAR vs. hypoxia (Supplementary Fig. 1e). Since an increase in ROS is associated with an increased HIF-1α-mediated response to hypoxia^27^^,^ we further investigated the downstream signaling cascade. As ROS affect the phosphorylation-dependent activity of the mitogen-activated protein kinase p38, which in turn influences HIF-1α stability^28^, we hypothesized that the increase in ROS levels in the presence of CAR is accompanied by a change in p38 activity. Indeed, phosphorylation of p38 was increased in the presence of CAR upon hypoxia (Fig. 2). Under normoxic conditions, CAR did not affect p38 phosphorylation, and total levels were unchanged (Supplementary Fig. 2). Together, these data suggest that CAR increases ROS-dependent oxidative stress, which in turn can stabilize HIF-1α via p38 activation upon hypoxia.

**Fig. 3:**
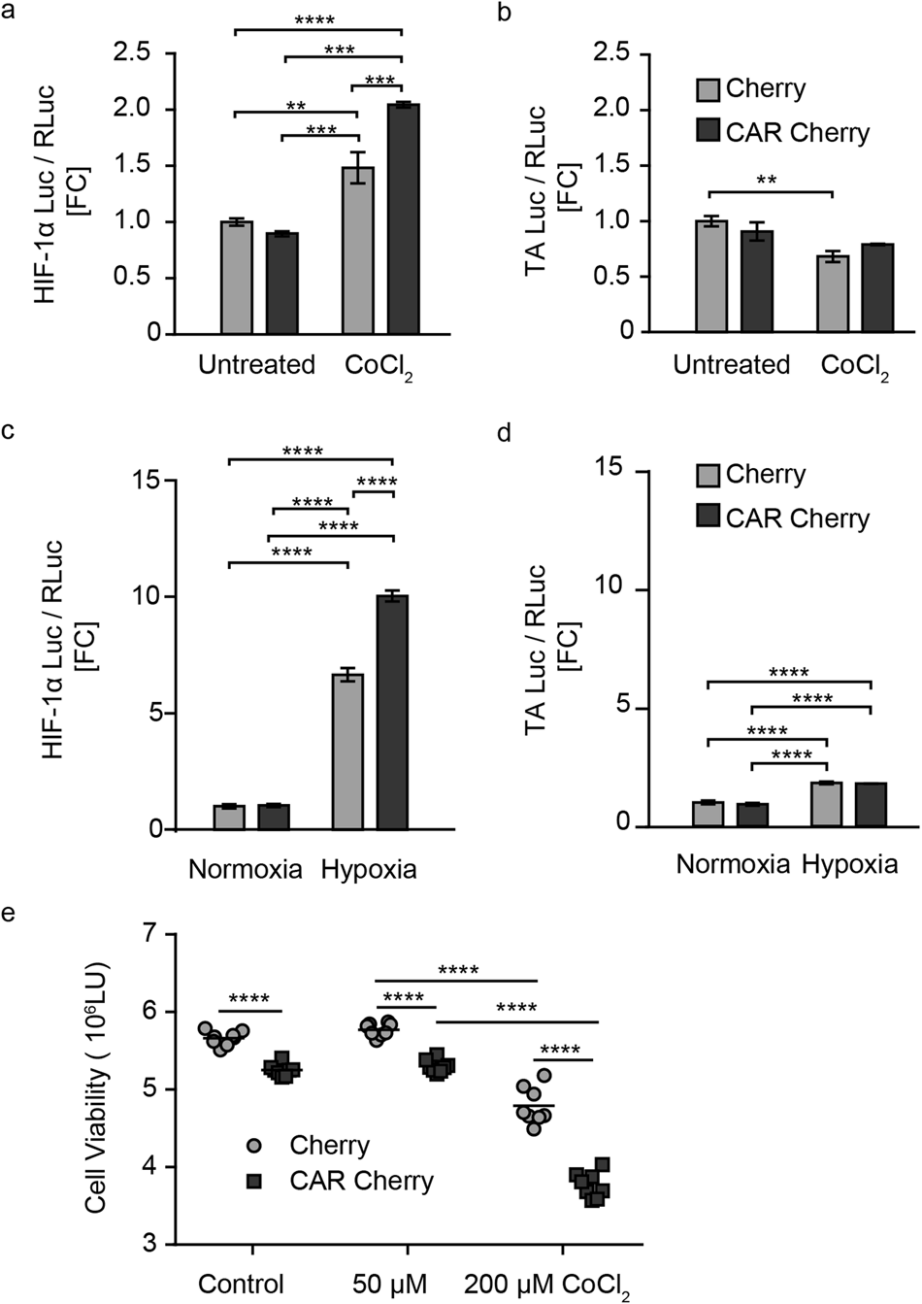
CAR regulates HIF-1α activity in vivo and in vitro. **a** Luciferase assay in HEK 293 cells cotransfected with HIF-1α Luc (**a** and **b**) or TA Luc reporter (**c**, **d**), RL-TK (internal control_Renilla Luciferase), and CAR Cherry or Cherry (*n* = 4). Twenty-four hours after transfection, the cells were either untreated or treated with 50 µM CoCl_2_ to induce chemical hypoxia (**a**, **b**) or with a gas mixture of 95% N_2_ and 5% CO_2_ to induce hypoxia for 10–12 min (**c**, **d**). Two-way ANOVA with Bonferroni’s post-test. *p < 0.05, **p < 0.01, ***p < 0.001 and ****p < 0.0001. **e** Cell viability after chemical hypoxia was reduced with higher concentrations of CoCl_2_ and overexpression of CAR versus the Cherry control. Two-way ANOVA with Bonferroni’s post-test. ***p < 0.001, **p < 0.01 and *p < 0.05 (*n* = 6).

To verify this proposed connection to HIF-1α levels downstream of p38, we evaluated the effect of CAR on HIF-1α using a dual luciferase reporter in HEK293 cells during chemical and physical hypoxia. During chemical hypoxia, HIF-1α activity was increased in the cells overexpressing CAR Cherry compared to the Cherry-transfected cells (Fig. 3a). In the TA luciferase (control)-transfected cells, there was no significant difference in transcription activity between the CAR Cherry and Cherry constructs under normoxic or hypoxic conditions. The reduced reporter activity in response to CoCl_2_ suggests that we might even underestimate the HIF-1α response (Fig. 3a, b).

The response to physical hypoxia was more pronounced than the response to chemical hypoxia, with >5-fold increased HIF-1α reporter activity in controls vs. a 10x increase in CAR Cherry-transfected cells (Fig. 3c). There was no difference in the luciferase activity between the groups and a negligible hypoxia-induced increase in the control luciferase reporter (Fig. 3d). Thus, independent of the type of hypoxic stimulus, CAR Cherry overexpression results in increased HIF-1α activity compared to Cherry controls. As the newly identified role of CAR upstream of the RhoA/ROS/HIF-1α signaling pathway is linked to apoptosis, we evaluated the effect of CAR on cell survival in a gain-of-function approach in HEK293 cells subjected to chemical hypoxia with CoCl_2_ (Fig. 3e). Forty-eight hours after transfection, CAR expression reduced cell survival under baseline conditions, and the effect was increased with CoCl_2_ treatment.

**Fig. 4:**
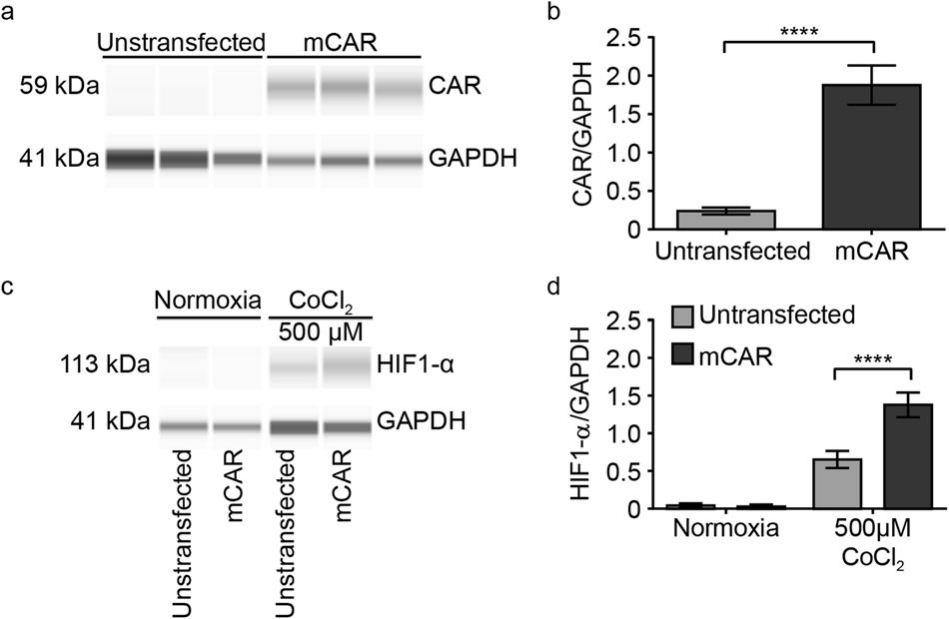
CAR overexpression in H9c2 cells enhances the Hif-1a response to chemical hypoxia. **a** WES blot and **b** quantification of the overexpression of mCAR in differentiated H9c2 cardiomyocytes; unpaired *t* test. ****p < 0.0001 (*n* = 9). Chemical hypoxia was induced by treatment with CoCl_2_ (500 µM) for 24 h, WES blot **c**, and quantification **d**, with two-way ANOVA and Tukey’s multiple comparisons test. ****p < 0.0001 (*n* = 9).

To characterize the activity of CAR in the RhoA/ROS/HIF-1α signaling pathway in a cardiac cell culture model, we transfected and selected cells and differentiated H9c2 myoblasts to cardiomyocytes (Fig. 4a, b; Supplementary Fig. 3). After induction of chemical hypoxia with 500 µM CoCl_2_, HIF-1α protein levels were increased in H9c2 mCAR transfected cells compared to the untransfected control (Fig. 4c, d). This enhanced hypoxia response in response to CAR overexpression was shared between HEK293 cells and H9c2 cardiomyocytes. In contrast, the hypoxia response was unchanged in isolated adult cardiomyocytes from control versus heterozygous CAR-deficient mice, as determined by the effect of 100 µM, 150 µM and 200 µM CoCl_2_ on 24 h viability (Supplementary Fig. 4). Lethality from 150 µM CoCl_2_ is prominent after 24 h, but CAR does not exert a protective role, possibly related to the cell dissociation that could interfere with CAR function as a cell-contact protein.

### Myocardial CAR depletion improves remodeling, cardiac function and survival after MI

Overexpression of CAR in cultured cells strongly suggested a role of CAR in hypoxia-related cell death signaling, as it also occurs upon an ischemic event in the heart. We therefore used an in vivo approach to determine whether loss of CAR could improve survival after MI and establish CAR as a therapeutic target.

**Fig. 5:**
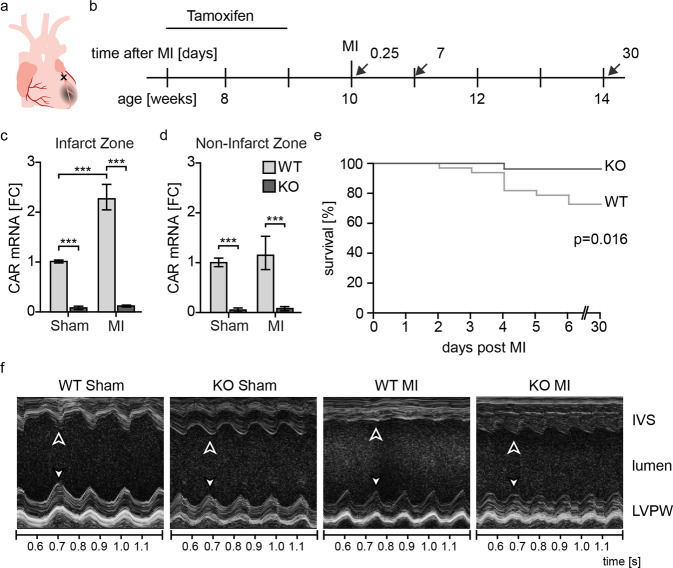
CAR deficiency improves survival and contractile function after MI. **a** Coronary artery ligation to induce MI. **b** Experimental design. Inducible heart-specific CAR KO mice at 7 weeks of age were injected with tamoxifen for two weeks. After one additional week, myocardial infarction (MI) was induced by ligation of the left anterior descending artery (LAD) at 10 weeks of age. Tissues were harvested after 0.25, 7 and 30 days. **c** CAR mRNA is upregulated in wild-type animals (WT) (light gray) but not in CAR-deficient animals (dark gray) after MI compared to the sham control. **d** Upregulation of CAR does not extend to the left ventricle (noninfarct zone). **e** Survival of CAR KO animals 7 days after MI was improved significantly compared to that of WT controls. **f** Sample traces of echocardiography M-mode with reduced mobility of the intraventricular septum (IVS) vs. unaffected left ventricular posterior wall (LVPW) in the WT after MI (flat line). Arrows indicate maximum contraction in systole. Quantification of echocardiography data is shown in Table 1. Data in **b** and **c** were analyzed for statistical significance with a two-way ANOVA, (*n* = 11). d was analyzed with a log-rank (Mantel‒Cox) test (*n* = 60).

To study the role of CAR in remodeling and scar formation after MI, we used inducible cardiomyocyte-specific CAR KO crossed with the tamoxifen-inducible mutant estrogen receptor fusion protein (MerCreMer) Cre line as described previously^12^. We injected tamoxifen over a period of 2 weeks in 7-week-old animals, and one week later, we induced MI by ligation of the left anterior descending artery (LAD) (Fig. 5a, b).

**Fig. 6:**
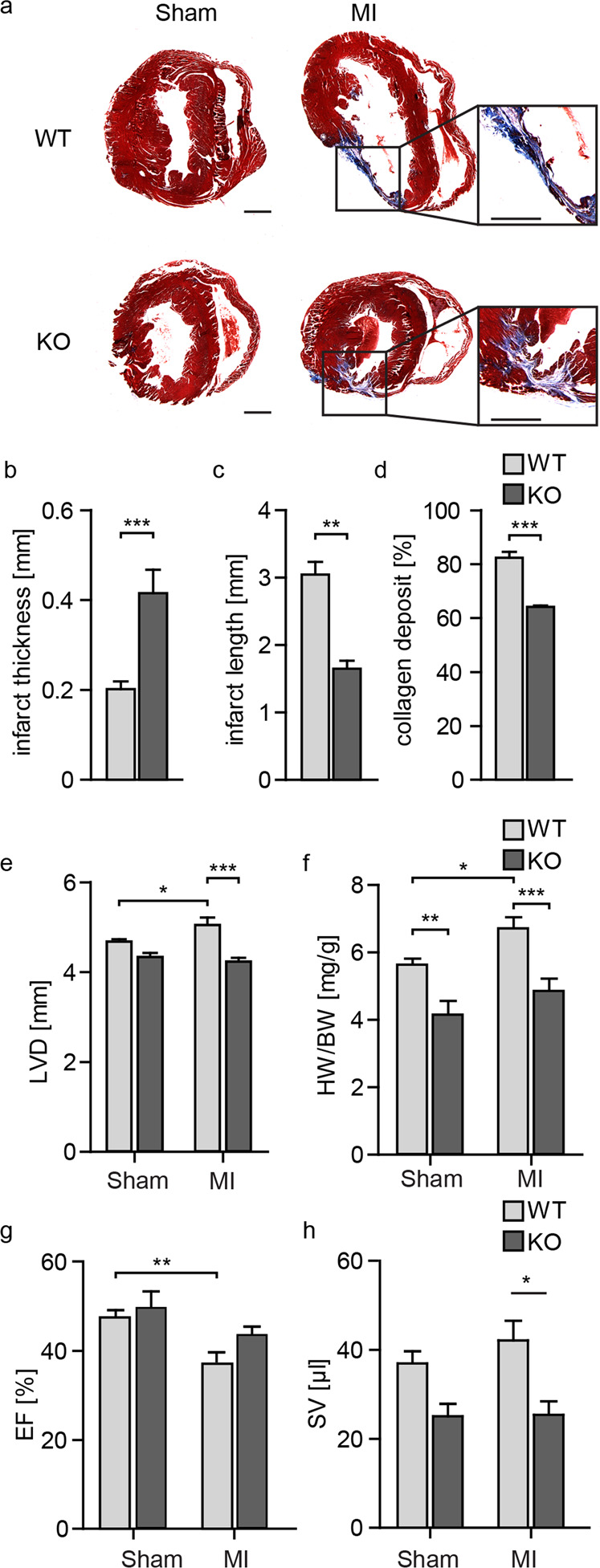
Loss of CAR reduces the size of the cardiac lesion post-MI and improves scar formation. **a** Representative trichrome staining of WT and KO heart sections 30 days post-infarction. Infarct width (**b**) and length (**c**) as well as collagen deposition (**d**) in the infarcted area were reduced in KO mice (*n* = 6, Student’s *t* test). **e** The left ventricular diameter in diastole (LVD) was only increased in the WT samples after MI. WT sham. **f** The HW to BW ratio indicates hypertrophy in WT animals. **g** The ejection fraction (EF) was significantly reduced in WT mice post-MI compared to sham control mice. **h** Stroke volume (SV) after infarction was significantly increased in WT mice compared to KO mice, which remained at control levels. Statistical significance was calculated using two-way ANOVA, *p < 0.05; **p < 0.01; ***p < 0.001. WT sham *n* = 3–8 per group.

Two days after coronary artery ligation, CAR expression was significantly induced in the wild type. This induction was confined to the peripheral region of the infarction, as expression in the remaining left ventricle remained unchanged. In the conditional knockout, CAR expression was reduced by approximately 90% (Fig. 5c, d). The remaining 10% relate in part to the expression of CAR in macrophages and endothelial cells, as revealed by the targeted analysis of single-cell data^29^. Survival rates after infarction were significantly different between the WT and KO mice: in the first week after infarction, ~30% of the WT animals died, while all cardiac KO animals survived this period except one (Fig. 5e). From the 7^th^ day after surgery, no additional deaths occurred in either group. Enhanced survival was associated with improved cardiac function 7 days post-MI as determined by echocardiography with improved motility of the interventricular septum (Fig. 5f), where the wall movement is represented as an almost straight line in the WT lesion compared to the undulating trace in the KO. At the structural level, CAR-deficient mice had lesions that were more compact with less collagen deposition than that in the WT hearts (Fig. 6a–d). Thirty days after infarction, the wild-type response to MI was eccentric hypertrophy with increased heart weight and left ventricular diameter, as well as reduced fractional shortening (FS) and ejection fraction (EF). In knockout animals, neither of these parameters was significantly altered, consistent with preserved cardiac function and the absence of secondary changes (Fig. 6e–h; Table 1).

**Table 1:** Improved cardiac function and morphology in CAR KO hearts 30 days post-MI.

	WT sham (*n* = 12)	KO sham (*n* = 7)	WT MI (*n* = 11)	KO MI (*n* = 9)
BW [g]	28.88 ± 1.00	28.50 ± 1.25	29.58 ± 0.96	29.82 ± 0.79
HW [mg]	162.30 ± 8.07	118.87 ± 15.55	198.34 ± 12.82*	143.18 ± 10.52
HW/BW [mg/g]	5.62 ± 0.20	4.14 ± 0.43*	6.70 ± 0.35*	4.84 ± 0.39
LVD diastole [mm]	4.66 ± 0.11	3.96 ± 0.21**	5.26 ± 0.14**	4.53 ± 0.11
LVD systole [mm]	3.51 ± 0.08	2.98 ± 0.19*	4.34 ± 0.13****	3.57 ± 0.09
FS [%]	24.72 ± 0.73	24.88 ± 1.98	17.64 ± 0.86****	21.01 ± 1.04
EF [%]	48.81 ± 1.35	49.86 ± 3.49	36.95 ± 1.75***	42.59 ± 1.94
SV [μl]	35.01 ± 2.33	24.24 ± 2.18	39.56 ± 3.78	26.13 ± 2.41
HR [bpm]	438.33 ± 14.18	506.14 ± 10.30**	496.64 ± 11.94*	520.44 ± 18.36***
CO [ml/min]	15.17 ± 0.86	12.35 ± 1.31	19.55 ± 1.92	13.73 ± 1.54

Two-way ANOVA.

*BW* body weight, *HW* heart weight, *LVD* left ventricular diameter, *FS* fractional shortening, *EF* ejection fraction, *SV* stroke volume, *HR* heart rate, *CO* cardiac output.

*p < 0.05; **p < 0.01; ***p < 0.001; ****p < 0.0001.

### Improved remodeling in CAR-deficient hearts after MI is associated with differential regulation of genes related to growth, apoptosis, and inflammation

**Fig. 7:**
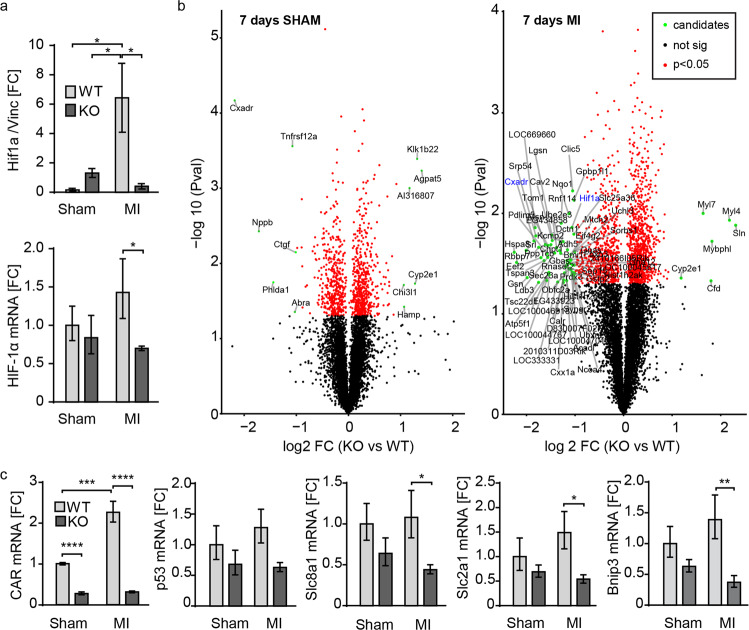
Downregulation of HIF-1a and target genes in CAR KO 7 d post-MI. **a** Quantification of HIF-1α protein induction 7 days post-MI normalized to the vinculin-loading control. WT sham (*n* = 3), WT MI (*n* = 4), KO sham (*n* = 5), KO MI (*n* = 4) and qRT- PCR analysis of mRNA from left ventricle for HIF-1α, sham (*n* = 4 per group) and MI (*n* = 5 per group). **b** RNA from left ventricles 7 days after infarction was isolated and analyzed in an Illumina Chip-Assay. Volcano plots of the log2-fold change in gene expression levels (x-axis) plotted against the –log10 *p* value (y-axis) comparing CAR KO with WT 7 days post-sham surgery (left) and MI (right). WT *n* = 3 per group; MI *n* = 5 per group. Significant genes (*p* < 0.05) are in red; regulated genes (absolute log2-fold changes >1 and *p* values <0.05) are in green. FC fold change. **c** qRT‒PCR analysis of mRNA from the left ventricle for CAR and the HIF-1α target genes p53, Slc8a1, Slc2a1, Bnip3. Sham (*n* = 4 per group) and MI (*n* = 5 per group); 18S was used as a housekeeper to normalize and calculate the fold change (FC) relative to the WT sham group. The bar graphs represent the mean ± SEM; two-way ANOVA with Bonferroni’s post-test. *p < 0.05; **p < 0.01. WT (*n* = 3 per group), WT MI *n* = 5, KO MI *n* = 5.

To evaluate the connection between the improved survival of the CAR KO mice post-MI and the Rho/Rock and/or HIF-1a pathway, we examined HIF-1α expression at the protein level as well as at the mRNA level and found that HIF-1α was only upregulated in WT but not CAR KO animals 7 days after MI (Fig. 7a).

Furthermore, an Illumina gene-chip analysis of left ventricular tissue 7 days after MI or sham surgery comparing WT and KO hearts was performed. The results revealed strong differential regulation of genes associated with metabolism, hypoxia response, and G-protein signaling (Fig. 7b; Supplementary Fig. 5). The strongest regulator between KO and control was CAR itself, as expected after efficient induction of the KO. The largest shift 7 d after MI in the CAR-KO vs. WT mice resulted from the blunted upregulation of >20 genes, including hypoxia-inducible factor-1 alpha (HIF-1a), a crucial transcription factor involved in angiogenesis, glucose metabolism, and apoptosis^30^. Expression of its target genes Slc2a1, Slc8a1, Bnip3 and p53 was reduced in the KO tissue compared with the WT tissue after MI, the latter not significantly (Fig. 7c).

Differentially regulated genes were associated with hypertrophic signaling, hypoxia, and apoptosis, as revealed by Gene Ontology analysis (Supplementary Fig. 5). This analysis includes regulation of GTPase activity and the mitogen-activated protein kinase (MAPK) pathway. As Erk1/2 was not significantly regulated (Supplementary Fig. 6), we focused our analysis on apoptosis and GTPase signaling pathways with RhoA and ROCK kinase, both linked to ischemic damage^19^. Downstream of RhoA/Rock signaling, we evaluated the expression of phosphatase and tensin homolog (PTEN), as it is increased post-MI and impairs cardiac dysfunction^31^. PTEN levels were indeed elevated post-MI in wild-type but not in CAR KO hearts (Supplementary Fig. 6d). Thus, CAR deletion in the mouse heart is indeed associated with inhibition of the RhoA/ROCK signaling cascade, which can contribute to the increased survival in these animals post-MI.

## Discussion

Current treatment of MI primarily targets the vasculature to enable early reperfusion, while the myocardium receives less attention^32^. In part, this issue is related to our insufficient understanding of the molecular pathology, exemplified by the local induction of CAR after MI^15^. Thus, elevated CAR levels in the border zone might be a beneficial reaction to contain the lesion or contribute to the pathogenesis of MI, warranting a diametrically opposite therapeutic approach.

To derive the hypoxia signaling network connected to CAR, we evaluated links between HIF-1α, RhoA, and ROS. Among these is PTEN, a tumor suppressor protein that contributes to apoptosis in ischemic cardiomyocytes via RhoA/ROCK signaling^19^. We found that PTEN protein levels were elevated 7 days post-MI in WT but not in CAR KO hearts, suggesting that PTEN might contribute to ischemic injury in the presence of CAR (Supplementary Fig. 6d). Consistent with our findings, pharmacological inhibition of PTEN limited infarct size and improved left ventricular function post-MI via an increase in ERK1/2- and AKT-dependent eNOS activity^33^. As PTEN is downstream of RhoA, its reduced expression might reflect the deregulation of GTPase activity in CAR KO mice (Supplementary Fig. 6).

Increased ROS levels during hypoxia activate P38 and thus stabilize HIF-1-α^28^. During hypoxia, ROS levels were significantly increased based on the reduction in the final electron acceptor (O_2_) for the electron transport chain in the mitochondria during oxidative phosphorylation^27^. We recapitulated the complete pathway in cells overexpressing CAR, as downstream of ROS, p38 and HIF-1α were activated under hypoxic conditions with increased HIF-1α reporter activity after both physical and chemical hypoxia.

ROS can directly and indirectly regulate Rho GTPases and thus influence actomyosin organization as well as trigger downstream activity of MAPKs such as p38^28,34^. RhoA is a GTPase regulated by extracellular matrix proteins, occludins, and cadherins, a class of cell contact proteins whose localization depends on CAR expression^35,36^. Several cell-contact proteins, such as tight junction proteins (occludins) and adherens junction proteins (cadherins), as well as extracellular matrix (ECM) proteins, induce the activity of small GTPases to regulate tissue development, wound healing and contractility^37,38^. CAR expression indeed increased RhoA activity, as determined by independent colorimetric and pulldown assays. As RhoA and HIF-1α act in a feed-forward loop during hypoxic conditions, we suggest that they jointly mediate the CAR-dependent response to ischemia^39^.

We not only validated the role of CAR in the ischemia response in the cardiomyocyte cell line H9c2 but also in vivo. To evaluate the role of CAR in MI, we subjected our heart-specific CAR knockout mouse to coronary artery ligation and used survival, cardiac function, and remodeling as a readout. In wild-type mice, CAR expression was significantly increased in the border zone of the infarction but not in the unaffected myocardium, similar to experimental MI in the rat^15^. Preventing this response in the heart-specific knockout resulted in improved survival in the first week after infarction. In this acute phase, cardiac rupture significantly contributes to mortality, with only 1/3 of affected patients surviving^40^. CAR-deficient hearts after MI are not as enlarged, the fibrotic area is more contained, and wall thickness at the site of infarction is increased, which would be consistent with a lower predisposition to cardiac rupture. Enhanced remodeling is associated with improved contractile function (increased ejection fraction) and thus reduced compensatory hypertrophy.

As a risk factor, loss of CAR might predispose patients to ischemia-induced ventricular fibrillation as we revealed a significant reduction in ventricular conduction velocity in heterozygous CAR deficient animals. Nevertheless, these animals were not prone to ventricular arrhythmias after induction of acute myocardial ischemia, unless provoked with additional pacing and carbenoxolone treatment^41^. The enhanced survival of our CAR deficient animals suggests that arrhythmia is not a limiting factor as the improved remodeling dominates the phenotypic outcome.

We and others have linked CAR to the regulation of connexin function and expression^11,12^, which could explain part of the protective phenotype. Both, pigs treated with the gap junction blocker heptanol^42^ and mice deficient in Cx43 had smaller lesions after MI^43^. If reduced cell-cell communication limits the spread of injury and the response to hypoxia, we would expect smaller lesions and a blunted induction of hypoxia inducible factor 1 alpha (HIF-1α) and its downstream targets. This is indeed the case (Fig. 7), but the transcriptomic changes we found after MI suggested a more complex molecular basis of the cardioprotection phenotype associated with loss of CAR. This includes a strong response in pathways that converge on the generation of ROS and the regulation of cell proliferation versus survival.

**Fig. 8:**
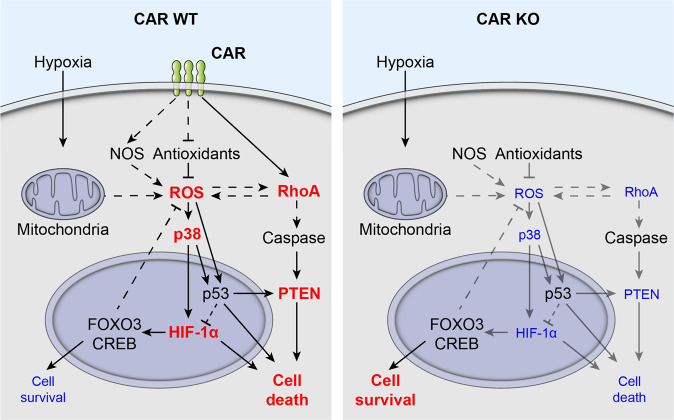
CAR interferes with RhoA and p38 to induce cell death in response to hypoxia. Schematic representation of the signaling pathways mediating the protective effect. ROS, reactive oxygen species. Dashed arrows indicate a multistep process. Red = increase; blue = decrease.

In summary, we found a novel role for CAR in exacerbating the response to hypoxia via a signaling network that includes the RhoA pathway, O_2_ tension, and ROS homeostasis to determine HIF-1α activity and cell survival during hypoxia (Fig. 8). Together with our in vivo data with improved survival and cardiac function of the heart specific CAR knockout after MI (Figs. 5 and 6; Table 1), these findings suggest CAR inhibition as a therapeutic approach to reduce myocardial damage and to improve the outcome of patients with MI. More broadly, since CAR interferes with hypoxia signaling, these findings suggest a role of CAR in several additional conditions associated with reduced oxygen availability, including cancer, stroke, sleep apnea and ischemic kidney disease.

## Supplementary information

Supplementary figures
