# Supplementary material for: CAR links hypoxia signaling to improved survival after myocardial infarction
Source: Exp Mol Med. 2023 Mar 20;55(3):643–52. doi: 10.1038/s12276-023-00963-9 (PMC10073142; doi:10.1038/s12276-023-00963-9)
Supplement: Supplementary file 1 — Supplementary figures [file 12276_2023_963_MOESM1_ESM.pdf]

Supplementary Information

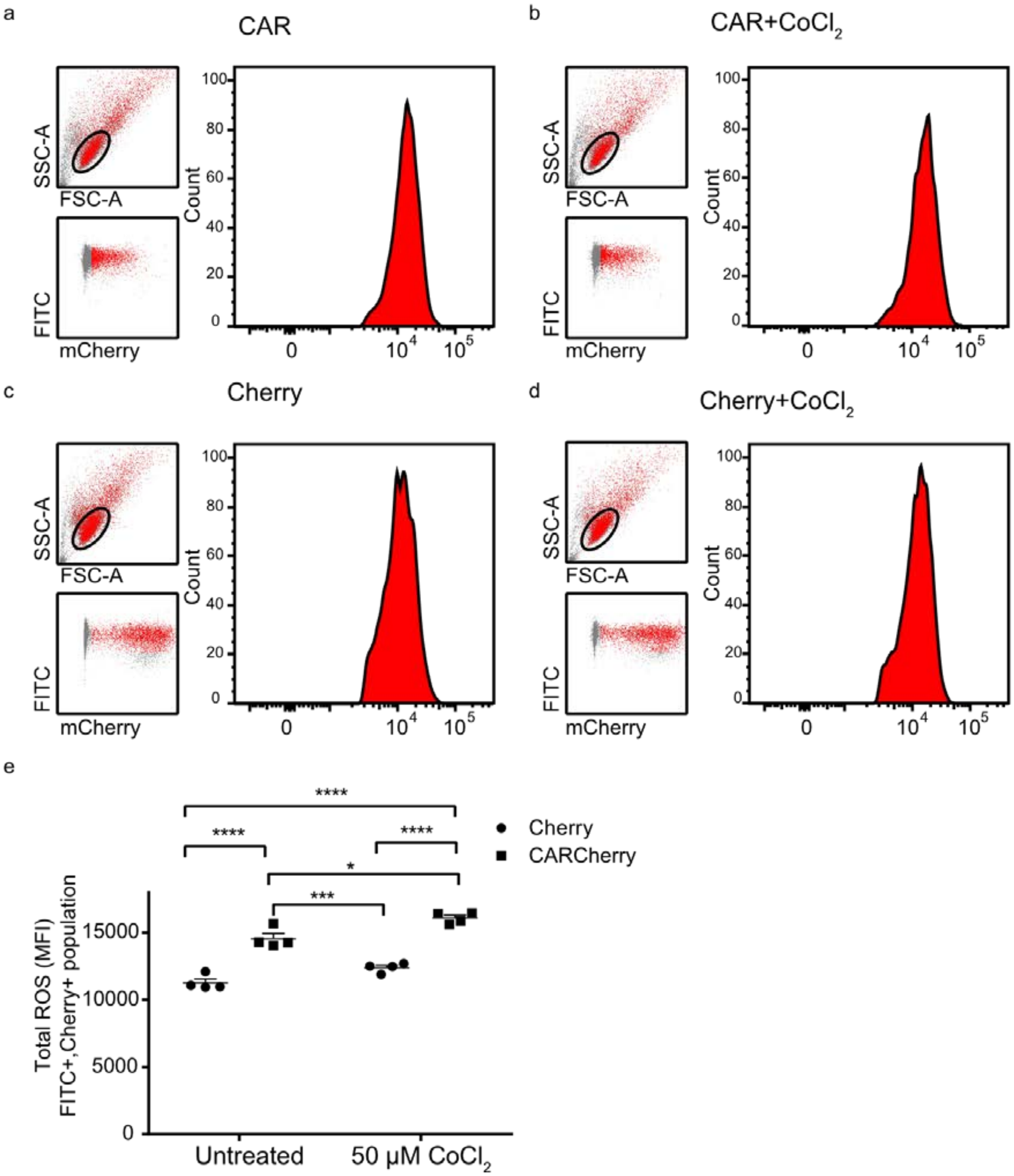

Supplementary Fig. 1. CAR overexpression increases sensitivity to chemical hypoxia. HEK 293 cells transiently transfected with Cherry or CAR Cherry with or without 50 µM $CoCl_2$ were loaded with oxidative stress dye for 30 minutes at 37ºC. **a-d)** The ROS level was determined by measuring the fluorescence (FITC) signal of the cherry positive cells using flow cytometry. **e)** CAR significantly increases ROS production and sensitivity to chemical hypoxia (n = 4). Two-way ANOVA with Bonferroni's post-test; *p < 0.05, ***p < 0.001 and ****p < 0.0001.

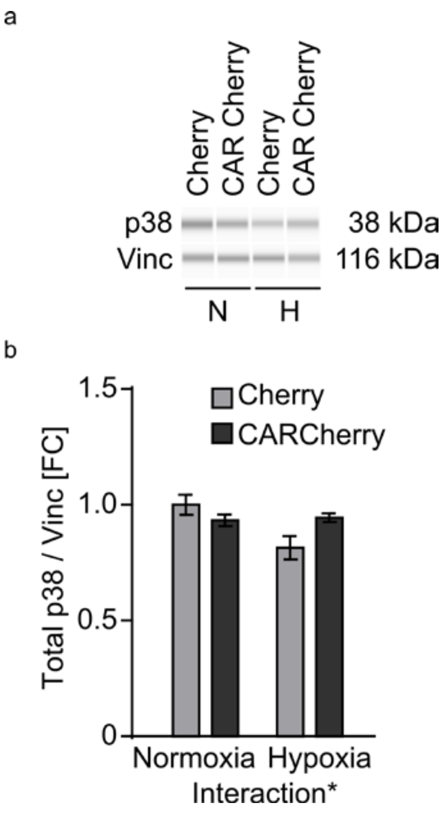

Supplementary Fig. 2. Total p38 protein expression is unchanged during hypoxia.

a) Protein levels in HEK cells overexpressing cherry or CAR Cherry for 48hrs cultured in normoxia (N) or hypoxia (H) for 24 hrs. **b)** Total p38 expression quantified by WES and normalized to vinculin levels. For statistical analysis two-way ANOVA with Bonferroni's post-test was used. n=6

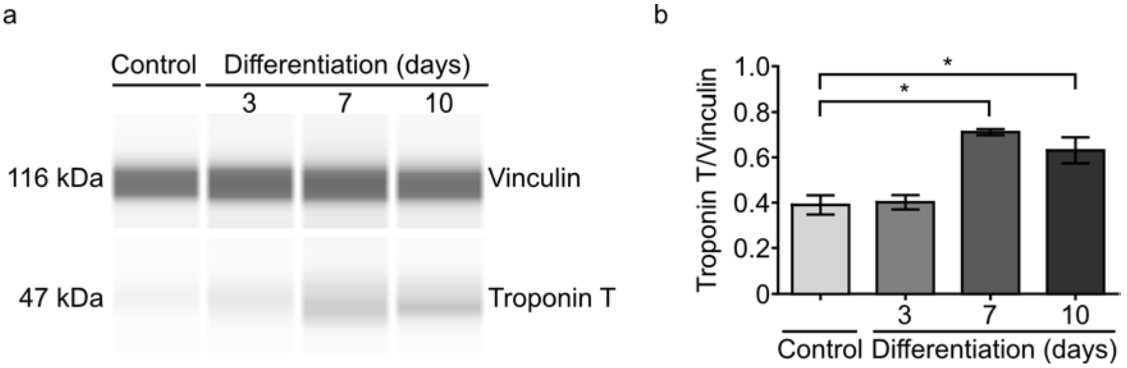

Supplementary Fig. 3. Differentiation of H9c2 myoblast into cardiomyocytes.

a, WES blot and **b**, quantification of the expression of Troponin T in H9c2 cells undifferentiated (n=5) differentiated with 1% FBS and 1 μM of RA for 3 (n=2), 7 (n=2) and 10 (n=6) days. The bar graphs represent Mean ± SEM with p* < 0.05 for One-way ANOVA.

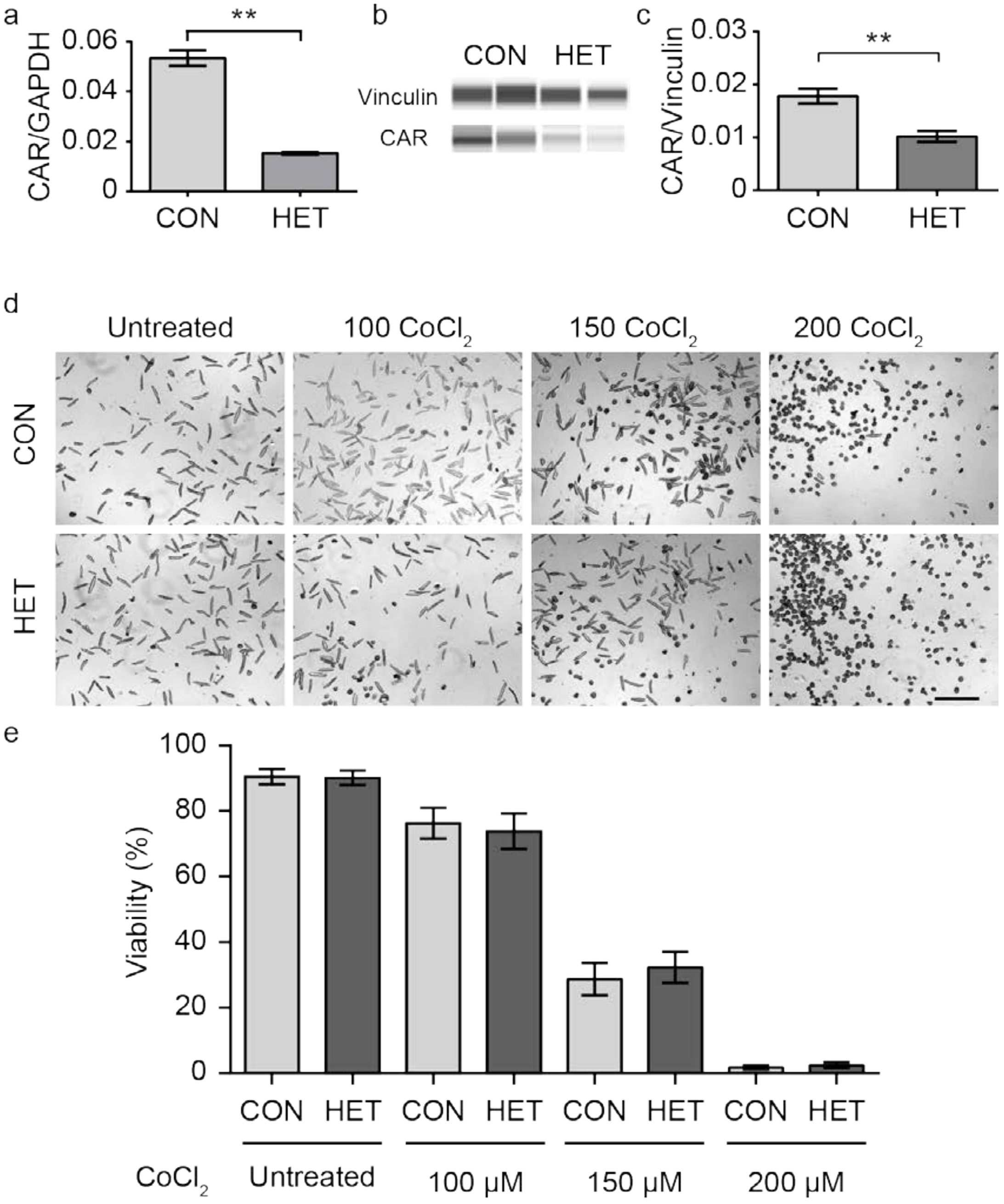

Supplementary Fig. 4. Viability analysis of CAR WT compared to CAR heterozygous cardiomyocytes after chemical hypoxia.

Reduction of CAR in heterozygous cardiomyocytes (HET) in hearts of Bl6CarE1 mice was verified by qRT-PCR (a) and Western blot (b, c) ($p^{**} < 0.01$, mean±SEM, n=6). Cardiomyocytes of WT and HET cardiomyocytes were untreated or treated by $CoCl_2$ (100 µM, 150 µM and 200 µM, 24 h) and viability was analyzed by counting rectangular, vital cells and dead cells (d, e) (mean ± SEM, n=9-12, scale bar 500 µm).

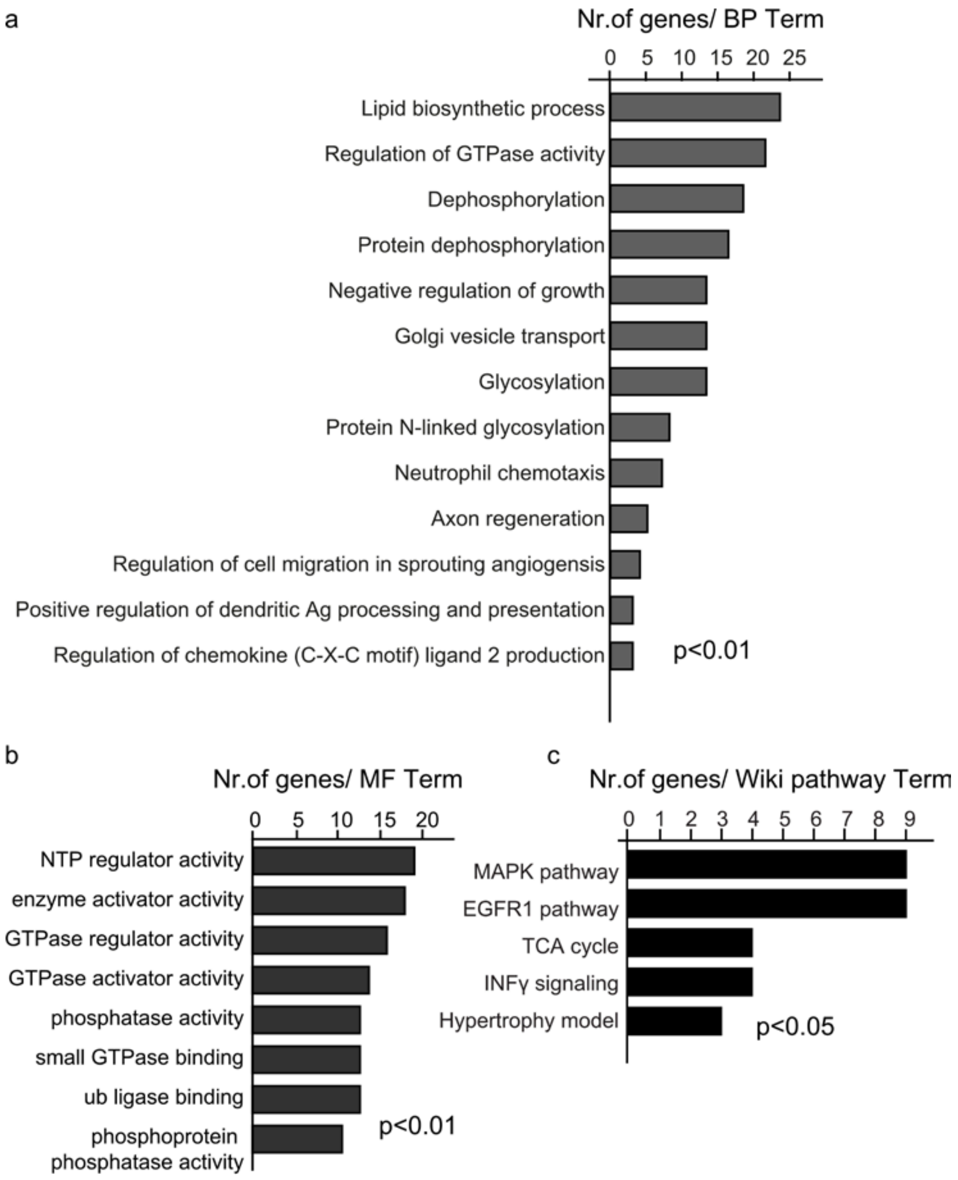

Supplementary Fig. 5. Gene ontology (GO) analysis of CAR KO gene expression data.

Genes regulated in the CAR KO with interaction p-value <0.05 after Two-way ANOVA analysis were inserted into Cytoscape software to perform gene ontology analysis using ClueGO plugin. Enriched terms with the indicated p-values from Bonferroni's post-test for either **(a)** biological process (BP), **(b)** molecular function (MF) or **(c)** Wiki pathways are depicted as bar graphs. The X-axis represents the number of regulated genes associated per term.

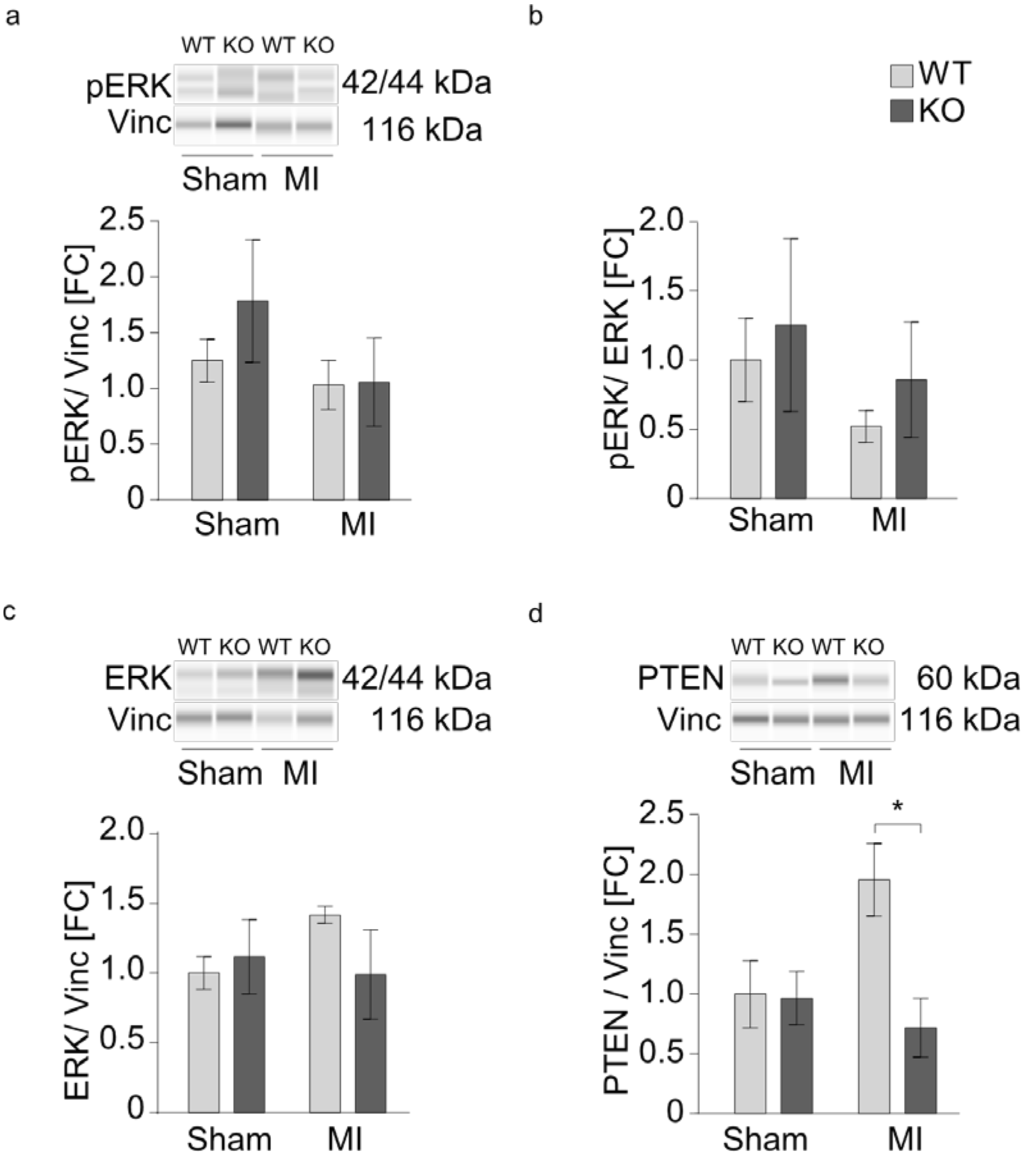

Supplementary Fig. 6. Verification of CAR KO gene expression data on protein level.

Quantification of whole protein lysates from left ventricle for pERK, total ERK, ratio of pERK/ total ERK **(a, b, c)** and PTEN (**d**) using WES (protein simple) normalized to vinculin. Sham n = 4, MI n = 5 for each WT and KO group. The bar graphs represent Mean ± SEM with $p^* < 0.05$, Two-way ANOVA with Bonferroni's post-test.
